# Phase and Structural Thermal Evolution of Bi–Si–O Catalysts Obtained via Laser Ablation

**DOI:** 10.3390/nano12224101

**Published:** 2022-11-21

**Authors:** Anastasiia V. Shabalina, Alexandra G. Golubovskaya, Elena D. Fakhrutdinova, Sergei A. Kulinich, Olga V. Vodyankina, Valery A. Svetlichyi

**Affiliations:** 1 Laboratory of Advanced Materials and Technology, Tomsk State University, 634050 Tomsk, Russia; 2 Research Institute of Science & Technology, Tokai University, Hiratsuka 259-1292, Japan; 3 Laboratory of Catalytic Research, Tomsk State University, 634050 Tomsk, Russia

**Keywords:** bismuth silicates, laser ablation in liquids, laser irradiation, thermal treatment, phase evolution, structural evolution, photocatalyst

## Abstract

Laser methods are successfully used to prepare complex functional nanomaterials, especially for biomedicine, optoelectronics, and heterogeneous catalysis. In this paper, we present complex oxide and composite nanomaterials based on Bi and Si produced using laser ablation in liquid followed by subsequent powder annealing. Two synthesis approaches were used, with and without laser post-treatment of mixed (in an atomic ratio of 2:1) laser-generated Bi and Si colloids. A range of methods were used to characterize the samples: UV-Vis diffusion reflection, IR and Raman spectroscopy, synchronous thermal analysis, X-ray diffraction, transmission electron microscopy, as well as specific surface-area evaluation. We also followed the dynamics of phase transformations, as well as composition, structure and morphology of annealed powders up to 800 °C. When heated, the non-irradiated series of samples proceeded from metallic bismuth, through β-Bi_2_O_3_, and resulted in bismuth silicates of various stoichiometries. At the same time, in their laser-irradiated counterparts, the formation of silicates proceeded immediately from the amorphous Bi_2_SiO_5_ phase formed after laser treatment of mixed Bi and Si colloids. Finally, we show their ability to decompose persistent organic molecules of Rhodamine B and phenol under irradiation with a soft UV (375 nm) source.

## 1. Introduction

There are several main phases that can crystallize in a system containing Bi, Si, and O components. Among them are metallic Bi and Si, oxides SiO_2_ and SiO_x_ (non-stoichiometric), different crystallographic polymorphs of Bi_2_O_3_ (α, β, γ, δ, ε), BiO_2_ and BiO_1,5_, and, finally, bismuth silicates, in particular, Bi_2_SiO_5_ (metasilicate), Bi_12_SiO_20_ (sillenite), and Bi_4_(SiO_4_)_3_ (orthosilicate, or eulytite). The latter bismuth silicates (BSOs) are attractive materials for a number of applications, such as sensors [1], capacitors [2,3], nanothermometers [4,5], acoustic devices and optical devices [6,7], fuel-cells [8], spatial light modulators [9], holographic grafting recorders [10], etc. Moreover, Bi–Si–O based materials find their application as bioactive materials [11], luminescent materials [12,13], and photocatalysts [14,15].

Sakamoto and coauthors mentioned that pure Bi_2_SiO_5_ could not be obtained by the conventional solid-state reaction from SiO_2_ and Bi_2_O_3_ [3]. Since metasilicate is metastable, it is the stable orthosilicate that is formed at the SiO_2_/Bi_2_O_3_ interface. Metastable metasilicate decomposes easily into sillenite and orthosilicate as follows: 16Bi_2_SiO_5_ → 5Bi_4_Si_3_O_12_ + Bi_12_SiO_20_ [3]. In contrast, Yiting et al. reported on [SiO_4_] tetrahedra and layers of [Bi_2_O_2_] molecules observed in a molten Bi_2_O_3_-SiO_2_ mixture [16]. Because this structure in the melt is very similar to that in Bi_2_SiO_5_, formation of the latter should be primarily expected [16]. Moreover, orthosilicate was reported not to nucleate and crystallize spontaneously without crystal seeds being available [16]. Hence, purposeful or at least controllable preparation of bismuth silicates seems to be quite challenging.

The phase composition of resultant products depends not only on the method and precursors used, but also on processing parameters applied. Mahmoud et al. used a hydrothermal method in a combination with sonication to obtain a bismuth silicates-based catalyst for oleic acid esterification with methanol [17]. They found that either Bi_4_Si_3_O_12_ or Bi_2_SiO_5_ was formed, depending on sonication time. Chen and co-authors varied the conditions of a template-free hydrothermal process (Bi/Si ratio, pH, and temperature) to obtain bismuth silicates-based materials with different compositions [18]. In particular, treatment at pH > 7 and 150 °C led to the formation of Bi_12_SiO_20_ and Bi_2_SiO_5_, while at 200 °C and 250 °C a mixture of Bi_4_Si_3_O_12_ and Bi_2_SiO_5_ or pure Bi_2_SiO_5_ was prepared, respectively [18]. Yiting et al. investigated the effect of the SiO_2_/Bi_2_O_3_ ratio on phases forming during and after cooling, demonstrating that at 30–45 mol.% of SiO_2_ the first crystallizing phase was Bi_2_SiO_5_ [16]. As the SiO_2_ content was elevated to 50 mol.%, phase SiO_2_ began to emerge in the product, while formation of phase Bi_4_Si_3_O_12_ was observed when more than 60 mol.% of SiO_2_ was used [16]. Wu et al. also changed the conditions of the Pechini sol-gel process to prepare different phases [19]. In particular, they varied the Bi/Si ratio, citric acid content and temperature, showing that an increase of heating temperature or time led to a gradual decrease in bismuth oxide content. This was explained by its reaction with Bi_2_SiO_5_ to form Bi_12_SiO_20_ [19]. At a fixed temperature, Bi_2_SiO_5_ was obtained by varying the Bi/Si ratio (at Bi/Si = 3). At the Bi/Si ratio between 3 and 10, the intensity of metasilicate peaks decreased while those of phase Bi_12_SiO_20_ increased. When the ratio achieved 10, only orthosilicate phase was found to crystallize.

Thus, such processing parameters as temperature, pH, additional treatment (sonication) and the Bi/Si ratio of precursors appear to be important for control of phase composition in the Bi–Si–O system irrespective of synthesis method applied. 

In our previous work [20] we obtained two Bi–Si–O-based nanomaterials using the method of pulsed laser ablation in water. This method is quite simple and environmentally friendly, requiring no complex laboratory equipment and toxic reagents, and permitting the production of species that cannot be obtained via conventional methods (like silica-containing soluble species, described in our previous works [20,21]). Moreover, for the first time we used an original synthetic approach. Briefly, we mixed fresh separately obtained colloids of Bi and Si species and either dried the mixture (BSO nanomaterial) or submitted it to additional laser treatment (BSO_hν nanomaterial). The obtained materials were studied, and only a trace amount of BSO phases was revealed (mainly in BSO_hν material). Nevertheless, some photocatalytic activity was found for both reported nanomaterials [20]. 

In the present work, we annealed the two nanomaterials prepared via laser ablation in water (samples BSO and BSO_hν) at seven different temperatures from 200 to 800 °C, carefully studying their thermal evolution in terms of morphology, phase and chemical composition. Structural and phase changes were investigated using a number of analytical methods, and photocatalytic properties of the materials were tested. For the first time, we obtained two groups of BSO-based materials (without and with additional laser post-treatment after their preparation) and revealed their detailed evolution caused by thermal treatment. Thus, the novelty and originality of the present study lies in: (i) a new approach to laser-based preparation of complex Bi–Si–O composites followed by systematic monitoring of phase and morphology evolution of nanoparticles during their annealing at gradually elevated temperatures; (ii) a thorough comparison of laser non-irradiated and laser-irradiated samples in the Bi–Si–O system during their heat-treatment; (iii) and finally in the evaluation of photocatalytic activity of the formed composites towards two distinct organic compounds with respect to the phase composition of the latter composites.

## 2. Experimental

### 2.1. Synthesis of Materials

**Figure 1:**
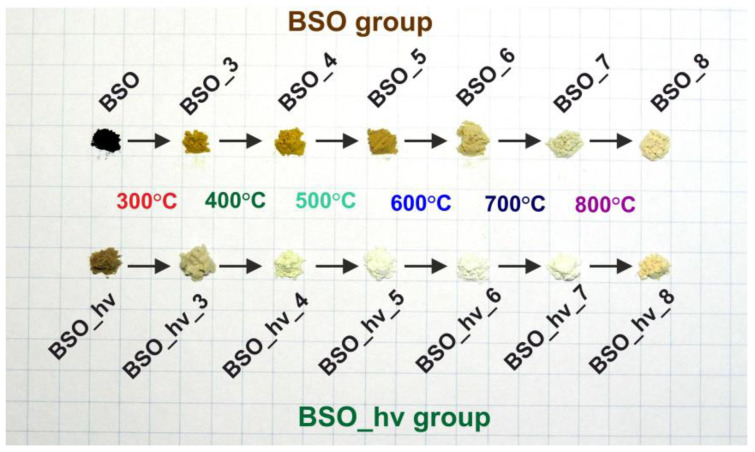
Photographs of initial and annealed powders studied. Images for samples annealed at 200 °C are not presented, as they did not differ from initial ones.

Two initial materials were synthesized according to previously reported procedures [20]. In particular, sample BSO was obtained via mixing of colloids generated by pulsed laser ablation of Bi (99.5% purity) and Si (99.99% purity) targets in distilled water (model LS-2131M-20 Nd:YAG laser, 1064 nm, 7 ns, 150 mJ, 20 Hz, LOTIS TII, Belarus) and subsequent drying. Its counterpart, sample BSO_hν, was obtained from the same mixed colloid that was additionally subjected to laser irradiation under the same conditions for 2 h and then dried. In the present work, these two initial materials were thermally treated to obtain two groups of samples (BSO group and BSO_hν group). The thermal treatment was performed as follows. A portion of initial powder was poured into a ceramic crucible and placed in a muffle furnace (SNOL 7.2/1100, Russia). Then, a target temperature of 200, 300, 400, 500, 600, 700, or 800 °C was reached and maintained for 4 h in air. The obtained material was marked in accordance with its initial sample and temperature of calcination (see Figure 1).

### 2.2. Characterization of Materials

Phase composition of the obtained materials was studied by X-ray diffraction (XRD) using an XRD 6000 diffractometer (Shimadzu, Japan). Phase identification was performed using the PDF-4 database.

TG and DSC analysis of powders was performed in Al_2_O_3_ (alundum) crucibles using synchronous thermal analysis instrument STA 449 F1 Jupiter (Netzsch, Germany) in the temperature region up to 1000 °C in dried air.

Vibrational states of obtained materials were studied by Raman spectroscopy in the range of 90–1000 cm^−1^ using a Raman microscope inVia-Basic (Renishaw, UK) with an excitation laser of 785 nm. FTIR spectra in the range of 340–4000 cm^−1^ were recorded using the technique of Frustrated Total Internal Reflection by a Tensor 27 spectrometer (Bruker, Germany) with MIRacle (PIKE, USA) universal ATR sampling accessory (Diamond/KRS-5 Crystal Plate).

Diffuse-reflectance spectra (DRS) were collected in the UV–Vis region using a Cary 100SCAN spectrophotometer (Varian, Australia) with a DRA-CA-30I accessory (Labsphere, USA). Then the following conversion was used (1):

*F*(*R*) = (1 − *R*)^2^/(2*R*) = α/*S*, (1)

where *F*(*R*)—Kubelka-Munk function, *R* is the diffuse reflection coefficient, α is the linear absorption coefficient, *S* is the linear scattering coefficient (it is assumed to be constant in the region of intrinsic absorption edge). The band gap was estimated from the edge of the absorption band by the Tauc method. For this, a graphical dependence was obtained in the following coordinates (2):

(α*hv*) = A(*hv* − *E_g_*)*^n^*, (2)

where *n* = 1/2 for direct-gap and *n* = 2 for non-direct-gap transitions.

In addition to the Tauc method, the band gap was estimated using the DASF (Derivation of Absorption Spectrum Fitting) method, which is discussed in greater detail elsewhere [22]. To estimate the band gap, a dependence was plotted in the following coordinates (3):

*d* ln(α/λ)/*d* (1/λ) = *n*/(*hv* − *E_g_*), (3)

A remarkable feature of the DASF method is the independence of the results from the type of transition [22]. An example of band gap estimation for sample BSO_4 is presented in Figure S1 (Supplementary Material).

Microstructure of materials was studied using transmission electron microscopy (TEM) on an HT-7700 instrument (Hitachi, Japan). Specific surface area of powders was estimated using BET method via low-temperature adsorption/desorption of N_2_ on a TriStar II 3020 tool (Micromeritics, USA). Prior to the adsorption stage, the samples were degassed in vacuum (10^−2^ Torr) at 200 °C for 2 h.

### 2.3. Photocatalytic Tests of Materials

The photocatalytic activity (PCA) of materials was evaluated by photodecomposition of aqueous solutions of Rhodamine B (Rd B, 5 µM) and phenol (50 µM) under irradiation with a light-emitting diode (LED) source with a wavelength of 375 nm. The total irradiation power on the sample was determined by a semiconductor detector PD300UV (Ophir, Israel) to be 51 mW. The catalyst loading was 0.5 g/L, and the volume of irradiated solution was 30 mL.

A dark stage was performed to establish the adsorption-desorption equilibrium in the system prior to irradiation. The concentration of Rd B in the decomposition tests was determined from its absorption spectra using a Cary 100 spectrophotometer (Varian, Australia). In the phenol decomposition tests, the concentration was found from fluorescence spectra using a spectrofluorometer CM2203 (SOLAR, Belarus). The apparent reaction rate constant *k_app_* (first-order reaction kinetics) was determined from the slope tangent from Formula (4):

ln(*C*_0_/*C*) = *k_app_ t*, (4)

where *C*_0_ is the initial concentration, *C* is the current concentration of target organics, and *t* is the reaction time.

In order to compare the results of PCA for different organics, the *k_app_* in h^–1^ calculated by Formula (4) was normalized to the organics’ concentration (in µM) and denoted as *K_app_*.

## 3. Results and Discussion

### 3.1. Phase Evolution during Annealing of Laser-Prepared BSO-Based Nanomaterials

Many previous works studied melts to investigate the crystallization of bismuth silicates in the Bi_2_O_3_-SiO_2_ system [16,23,24,25]. In this case it is quite easy to control the ratio of components and observe results. There are many reports on the synthesis of nanostructural silicates using sol-gel [3,19,26,27] or hydrothermal approaches [17,18], where a sufficiently good homogenization of the reaction mixture is also possible during material preparation. In this regard, the main feature of the system studied in this work was its initial inhomogeneity of component distribution. In our case, mixing did not occur during thermal treatment, and thus during heating, phase growth was determined by a local component ratio and by a diffusion propagation front. That is, at the nominal ratio Bi/Si = 2 used, local ratio values were different for different zones of the heated material. This can explain why the phase composition of our products might differ from those obtained by others.

**Figure 2:**
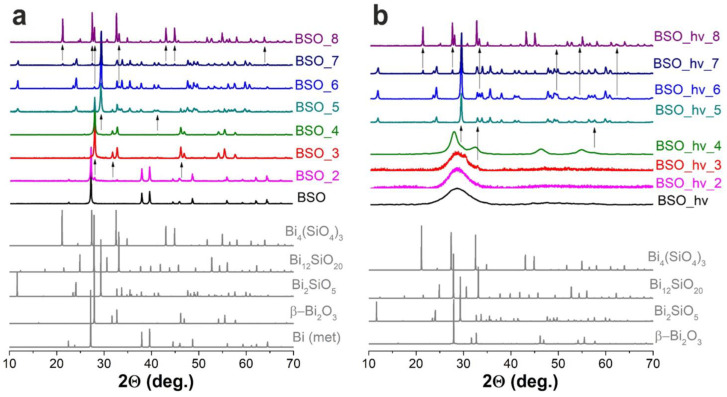
XRD patterns of non-irradiated samples BSO (**a**) and irradiated samples BSO_hν (**b**) before and after thermal treatment. Some evolution phase transitions are shown with arrows. PDF# base card numbers for known phases: 04-007-9968 (Bi); 04-015-6851 (β-Bi_2_O_3_); 00-036-0287 (Bi_2_SiO_5_); 01-080-9154 (Bi_12_SiO_20_); and 00-033-0215 (Bi_4_Si_3_O_12_).

**Figure 3:**
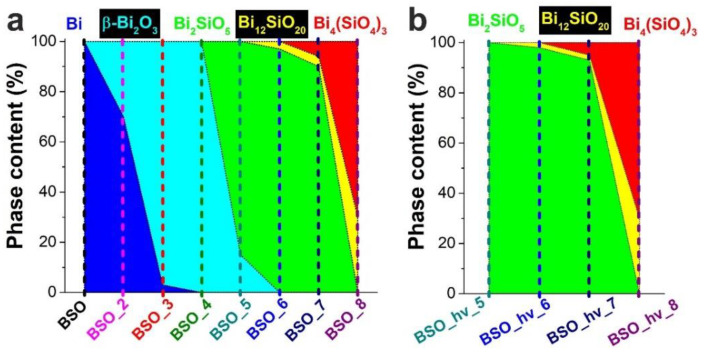
Phase composition of the samples and its evolution with thermal treatment. (**a**) Samples BSO, and (**b**) samples BSO_hν. Areas of phase presence are colored with same colors as their labels on the top.

The changes in phase composition of samples BSO and BSO_hν during thermal treatment were studied by XRD, with the recorded patterns being presented in Figure 2. Phase analysis was carried out, and detected phases are thoroughly marked in Figure S2 (Supplementary Material). Phase composition evolution for all the samples is schematically presented by colored maps in Figure 3 and is also tabled in Table S1 (Supplementary Material). In addition to XRD, to study the thermal phase evolution in the materials, other analytical methods were applied, such as TG, DSC, UV-vis, FTIR, and Raman spectroscopy. Moreover, color changes during heat treatment of powders are presented in Figure 1.

#### 3.1.1. Samples of the BSO group

##### Samples BSO and BSO_2

As was shown in our previous work [20], the initial sample BSO contained metallic Bi (PDF-4 #04-007-9968) covered with an amorphous Si-containing layer. The latter layer was concluded to protect metallic bismuth from oxidation or interaction with CO_2_ that could lead to the formation of carbonates and oxycarbonates during drying. 

**Figure 4:**
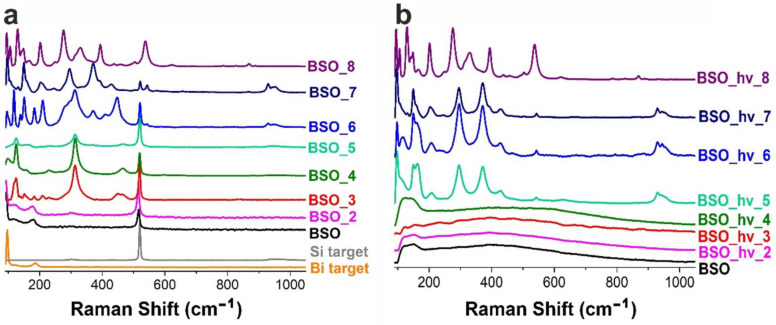
Raman spectra of the BSO (**a**) and BSO_hν (**b**) groups of materials.

Two bands in the Raman spectra of the samples observed at ~98 and 185 cm^−1^ in Figure 4a belong to vibrations of metallic Bi. The former is attributed to the first-order optical band of rhombohedral bismuth corresponding to non-degenerate A_1g_ phonon mode [20]. One more *E_g_* (doubly degenerate) expected at ~71 cm^−1^ is beyond the spectral range of the device used in the present work. The weak and broadened second-order band at 185 cm^−1^ consists of three overtones of similar frequencies [28]. The signals of metallic Bi were observed in the samples annealed at temperatures up to 200 °C. Moreover, as previously reported in work [20], the Raman spectrum of the initial BSO material contained a well-manifested peak of crystalline Si seen at ~520 cm^−1^. This signal was visible in the samples up to BSO_6 (Figure 4a), indicating survival of Si phase up to 600 °C. Note that this phase was not observed in XRD patterns due to its small amount.

As seen from the XRD data, heating up to 200 °C led to a partial oxidation of Bi particles and formation of oxide β-Bi_2_O_3_ (PDF-4 #04-015-6851). The Raman spectrum of sample BSO_2 is seen in Figure 4a to contain the above-mentioned signals of metallic Bi and crystalline Si, while first signs of phase β-Bi_2_O_3_ (band at ~310 cm^–1^) are already seen.

**Figure 5:**
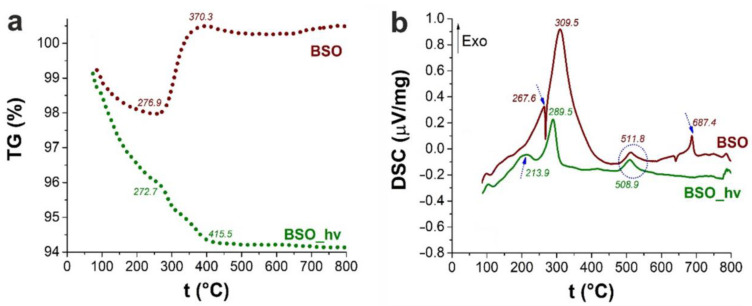
TG (**a**) and DSC (**b**) data for the BSO (brown color) and BSO_hν (green color) groups of materials.

**Figure 6:**
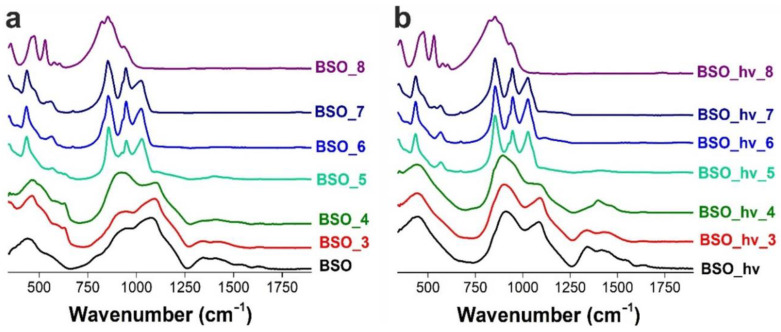
FTIR spectra of the BSO (**a**) and BSO_hν (**b**) groups of materials. Spectra for samples treated at 200 °C are identical to those of initial samples.

At the same time, TG results shown in Figure 5 demonstrate mass changes before 200 °C which are believed to be associated with oxidation processes and removal of physically adsorbed water and CO_2_, CO_3_^2−^ or HCO_3_^-^ from the surface °C [29]. The presence of these species is confirmed by the FTIR spectra in Figure 6a, where vibration bands of carbonates adsorbed on the particle surface are well seen in the range of 1300–1600 cm^−1^ [20]. The intensity of these bands decreases as the annealing temperature is elevated. A weak peak at 1640 cm^−1^ found in all of the spectra corresponds to the stretching vibration and bending vibration mode of hydroxyl coming from surface-adsorbed H_2_O molecules. It disappears upon thermal treatment and correlates with other water bands (O-H vibrations in the region of 3000–3500 cm^−1^, not shown here).

**Figure 7:**
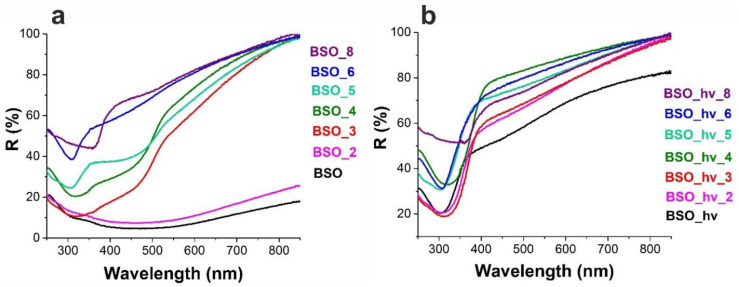
DRS of the BSO (**a**) and BSO_hν (**b**) groups of materials.

Both samples BSO and BSO_2 are seen in Figure 1 to be dark-brown. Their diffusion reflectance spectra only had wide featureless bands over the entire spectral range (Figure 7a). This can be explained by the presence of a metallic Bi phase. The beginning of β-oxide formation is not seen in the spectrum of sample BSO_2 because of a background associated with a large amount of metallic Bi.

##### Samples BSO_3 and BSO_4

A further elevation of annealing temperature to 300 °C was observed to result in further oxidation of metallic Bi, with almost pure β-oxide (97%) found in sample BSO_3 (Figure 3). For this sample, whose initial color was deep mustard, the formation of characteristic semiconductor edges of the absorption band in the region of ~500–550 nm is gradually observed in Figure 7a. It corresponds to the band of β-Bi_2_O_3_, which is a non-direct-gap semiconductor with a band gap *E_g_*~2.4 eV [30,31]. Moreover, sample BSO_3 demonstrates a very weak shoulder at 350 nm (Figure 7a). We assume that the shoulder can indicate the initial formation of bismuth metasilicate phase Bi_2_SiO_5_, a direct-gap semiconductor with *E_g_*~3.40–3.64 eV [28,30,32]. However, this phase is not yet visible in the XRD patterns, probably because of its trace amounts in the sample.

Thermal treatment at 400 °C was found to promote metallic Bi oxidation, giving rise to an almost 100% yield of β-Bi_2_O_3_. Below this temperature, no XRD signals of any Si-containing phases was found for the BSO group of materials.

The FTIR spectra of both samples BSO_3 and BSO_4 presented in Figure 6a demonstrate absorption in the range of 550–650 cm^−1^. The peak around 630 cm^−1^ belonging to the Bi-O stretching vibration in BiO_6_ octahedra [30,33] is well consistent with the bismuth oxide phase found by XRD.

Sample BSO_4, whose powder was initially of a deep mustard color (see Figure 1), had a UV-vis spectrum similar to that of sample BSO_3. However, its diffuse absorption is seen in Figure 7a to be lower in the visible region, which should be associated with a lower content of metallic Bi. The shoulder related to the initial formation of metasilicate in this sample is seen to be more pronounced in the spectrum.

According to the TG data, only above 400 °C does the mass of the sample seen in Figure 5a begin to stabilize at a value of 100.3% of the initial one. Prior to this, there is a decrease in weight (approximately by 0.5%) between 200 °C and 260–270 °C followed by a sharp increase in mass between 270 and 400 °C. The DSC data presented in Figure 5b demonstrate an endo-peak of metallic bismuth melting just around 270 °C [34] and then the most intense exo-peak around 310 °C. The latter peak may be attributed to the α-phase formation [35] and the α → β phase transition of Bi_2_O_3_ [36]. Moreover, one cannot completely exclude formation of metastable bismuth metasilicate in this temperature range [35].

Raman studies also confirmed the presence of β-oxide in samples BSO_3 and BSO_4, as a set of the three most intense peaks at 124, 313, and 464 cm^−1^, corresponding to well-known Bi-O stretching vibrational modes, which is well seen in Figure 4a [37].

##### Samples BSO_5, BSO_6, BSO_7 and BSO_8

As seen in Figure 6a, upon further annealing of the samples, a set of additional narrow vibrational bands appeared in the FTIR spectra of samples BSO_5, BSO_6 and BSO_7, which are characteristic of bismuth metasilicate Bi_2_SiO_5_. First of all, there are three intense bands at 855, 945, and 1030 cm^−1^ that are assigned to the stretching vibration mode of Bi–O–Si bonds, isolated SiO_5_^6−^ groups, and to the stretching modes Si-O-Si of the SiO_4_ tetrahedral units, respectively [28,38,39]. In the longer-wavelength spectral region, there are bands of phase Bi_2_SiO_5_ at 432 and 565 cm^–1^ and a weak band at 675 cm^–1^, which are attributed, respectively, to the stretching vibration mode of Bi-O bonds, to bending (SiO_4_)^4–^ groups, and presumably to the bending vibrations of the Si-O bond [38,39].

In the Raman spectrum of the BSO material calcinated at 500 °C (of a mustard color, Figure 1), the signals of oxide can be seen, similar to those in the spectra of samples BSO_3 and BSO_4 (Figure 4a). However, in the UV-vis spectrum of sample BSO_5, the shoulder belonging to the metasilicate formation increases (Figure 7a). At the same time, the first XRD signals of a joint Bi–Si phase formation were only observed in the samples from the BSO group that were thermally treated at 500 °C. In sample BSO_5, about 85% of bismuth metasilicate (PDF-4 #00-036-0287) was found to be formed from β-Bi_2_O_3_. Thus, significant interaction of Bi and Si components was only observed in non-irradiated BSO materials after their heating at 500 °C and above.

The Raman spectrum of sample BSO_6 (powder of a cold beige color, Figure 1) contains characteristic modes of vibrations of Bi_2_SiO_5_ (Figure 4a)**.** The peaks located at 150 and 543 cm^–1^ are related to the vibrations of Bi atoms, and the peaks at 206 and 372 cm^–1^ are respectively attributed to the rocking and whirling of [SiO_4_] groups. The peak at 297 cm^–1^ corresponds to the stretching vibration of Bi-O bonds, while the peak at 431 cm^–1^ is assigned to the vibrations of oxygen atoms in the [BiO_4_] group. The other two peaks located at about 930 and 947 cm^–1^ are ascribed to the stretching vibration of Si-O bonds [28]. All this is well consistent with XRD observations which registered metasilicate as the dominant phase in the sample heated at 600 °C (Figure 3a). Further increase of annealing temperature was found to lead to the complete oxidation of crystalline silicon, so that the peak at ~520 cm^–1^ disappeared. At the same time, vibrations belonging to sillenite Bi_12_SiO_20_ [40] and orthosilicate Bi_4_Si_3_O_12_ [39,41] emerged in Figure 4a. Three broad and well-pronounced bonds are seen in a complex spectrum of sample BSO_7: at 278 (O^2-^ “breathing” and weak Bi–O1 rocking), 328 (Bi–O1 rocking and weak O^2-^ “breathing”), and 540 cm^−1^ (“breathing” of O1 atoms) [40]. Two intense peaks characteristic of orthosilicate seen at ~200 and 395 cm^–1^ belong to lattice vibrations and modes of [SiO_4_] units, respectively [39].

Diffraction patterns show that another joint Bi–Si phase (sillenite, Bi_12_SiO_20_, PDF-4 #00-037-0485) began to form after 600 °C, while phase Bi_4_(SiO_4_)_3_ (PDF-4 #00-035-1007) first emerged above 700 °C. The DSC curve in Figure 5b exhibits exo-peaks at 510 and 690 °C corresponding to the β-Bi_2_O_3_ to Bi_2_SiO_5_ transition [35] and to the recrystallization of metastable phase Bi_2_SiO_5_ to stable sillenite Bi_12_SiO_20_ [42], respectively. Above 700 °C, both Bi_2_SiO_5_ and Bi_12_SiO_20_ are known to interact with SiO_2_, resulting in the stable phase of Bi_4_(SiO_4_)_3_ [29]. In the UV-visible spectra in Figure 7a, the edge at 350 nm (bismuth metasilicate) is well-seen for sample BSO_6. However, a pronounced adsorption at 500 nm belonging to β-Bi_2_O_3_ disappears in this sample, while the long-wavelength edge shifts to 450 nm, which can be associated with the absorption of the indirect-gap semiconductor bismuth orthosilicate Bi_4_Si_3_O_12_ with *E_g_*~3.0–3.3 eV [43].

As seen in Figure 3, bismuth metasilicate remains as the main phase even after annealing at 700 °C, only disappearing at 800 °C. According to XRD, sample BSO_8 heat-treated at 800 °C contained Bi_4_(SiO_4_)_3_ as the main phase (70%) mixed with sillenite (30%), which is confirmed by its UV-visible spectrum where the band at 450 nm becomes more pronounced (Figure 7a). In the FTIR spectrum of sample BSO_8 (Figure 6a), formation of the high-temperature phase of bismuth orthosilicate Bi_4_Si_3_O_12_ is associated with the bands at 480, 535, 820 and 885 cm^−1^. In the Bi_4_Si_3_O_12_ structure, Bi and Si atoms are arranged in octahedral [BiO_6_] units. Therefore, the peaks seen in Figure 6a near 482 cm^–1^ were assigned to the vibration of BiO_6_ octahedra and those near 535 cm^–1^ were assigned to the vibration band *ν*_2_ of (SiO_4_)^4−^ [39]. The band near 820 cm^−1^ was related to the symmetrical vibration of SiO_4_ groups, and that at 885 cm^−1^ was associated with the vibration of the Bi–O–Si bond [44]. They all started emerging at 700 °C, becoming well-seen for sample BSO_8. In addition, the peak observed at 606 cm^−1^ for sample BSO_8 can be ascribed to cationic vibrations in the network or Bi–O vibration of bismuth sillenite Bi_12_SiO_20_ [45]. The samples heat-treated at the highest temperatures, i.e., BSO_7 and BSO_8, were of a light-beige and cream color, respectively (Figure 1).

**Figure 8:**
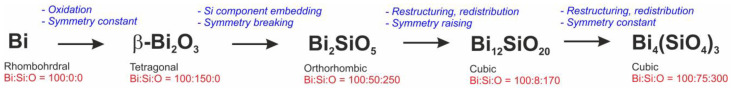
Scheme of phase transformation during thermal treatment of samples.

Schematically, therefore, the phase evolution for non-irradiated sample BSO as it was annealed at varied temperatures up to 800 °C can be presented as follows: Bi → β-Bi_2_O_3_ → Bi_2_SiO_5_ → Bi_12_SiO_20_ → Bi_4_(SiO_4_)_3_, which is also presented in Figure 8. The change of component ratios and the symmetry of crystallographic systems occurring during thermal evolution indicates that active interaction between Bi and Si components is stimulated by heating.

#### 3.1.2. Samples of the BSO_hν Group

It is noteworthy that the TG data for sample BSO_hν are very different from those for its non-irradiated counterpart (Figure 5a). Up to 200 °C, the weight loss for the irradiated sample is seen in Figure 5 to be 3.5% (against ~2% for the non-irradiated material). In this region, similar to sample BSO, adsorbed species are removed (DSC data, Figure 5a). Next, the peak seen at 214 °C on the DSC curve can probably be attributed to the formation of silanol groups on the surface of bismuth, resulting from dehydration and possibly partial dehydroxylation processes [46,47]. The silica component in this sample behaved differently from that in sample BSO, as was previously shown elsewhere [20]. Further heating of the sample leads to the most intense peak appearing around 290 °C (Figure 5b). It is narrower than that of non-irradiated material and can be attributed to the formation of metastable bismuth metasilicate [35]. Nonetheless, the possibility of bismuth oxide formation cannot be excluded.

According to XRD, however, all the samples of this group that were annealed at temperatures below 500 °C demonstrated an amorphous structure (see Figure 2). The broad amorphous peak centered at 28° and easily seen for the first three samples in Figure 2 (black, magenta, and red patterns), was slightly shifted and acquired a more pronounced form after heating at 400 °C (olive pattern in Figure 2). At this temperature, three more broad and low-intensity peaks appeared, pointing at somewhat increased crystallinity of the material. This diffraction pattern coincided better with that of phase β-Bi_2_O_3_, while some features (marked with arrows in Figure 2b) that appeared even at 300 °C may belong to bismuth metasilicate. 

The samples from BSO_hν to BSO_hν_4 do not exhibit well-pronounced characteristic signals in Raman spectra (black, magenta, red, and olive spectra in Figure 4b). They provide a wide low-intensity band in the range of 120–150 cm^−1^ that can be attributed to heavy-metal ion vibrations, i.e., those involving Bi^+3^ cations in BiO_3_ pyramidal units, or lattice vibrations in unformed bismuth silicate [20,28]. Another wide structureless band between 200 and 600 cm^−1^ can manifest the beginning of bismuth silicate formation. 

In the FTIR spectrum of the initial non-annealed sample BSO_hν (Figure 6b), a shoulder is observed around 855 cm^–1^, which indicates the formation of the Bi-O-Si interface in the nanomaterial that was laser-irradiated as a post-treatment stage during its preparation [20]. This band is known to be related to the stretching vibration mode of Bi–O–Si bonds in the structure of bismuth metasilicate Bi_2_SiO_5_ [38]. It is also seen in the spectra of samples BSO_hν_3 and BSO_hν_4.

In the UV-visible spectra of the initial laser-irradiated sample BSO_hν (whose powder was sand-colored) and of samples annealed at lower temperatures (note that sample BSO_hν_3 was of beige color), one can observe a weakly pronounced shoulder around 500 nm (Figure 7b). This feature, similar to what was observed for non-irradiated samples, should be attributed to an early stage of β-Bi_2_O_3_ formation. However, the band at 350 nm, which was previously attributed to the formation of bismuth metasilicate [20], appears even in the spectrum of non-annealed sample BSO_hν and is present in all the samples annealed at temperatures up to 600 °C (Figure 7b). Moreover, for samples BSO_hν_2, BSO_hν_3 and BSO_hν_4 (all being light-yellow), it slightly shifts towards the shorter-wavelength region, and for samples BSO_hν_5 and BSO_hν_6 (both are of milky color) it is shifted back to longer wavelengths, corresponding to the position of this band in a non-annealed sample BSO_hν (Table 1). The reason for such shifts is probably related to some interactions between different phases forming in the samples and requires further studies.

According to the TG data exhibited in Figure 5a, similar to the non-irradiated sample BSO, the mass of the laser-irradiated material also stabilized after 400 °C. However, the latter sample exhibited a gradual mass decline, with the final value being ~94%. We assume that this is due to the absence of metallic bismuth in this sample, which transforms into oxide in the sample BSO upon heating, thus increasing its mass (see above). According to XRD data, after annealing at 500 °C, the sample turned to pure Bi_2_SiO_5_. This agrees well with Figure 5b where, in the DSC curve, the exothermic peaks at 508 and 573 °C manifest the stabilization of phase Bi_2_SiO_5_ [48]. The Raman spectra of the irradiated samples heated at 500 and 600 °C (Figure 4b) only contain the known vibrational bands of phase Bi_2_SiO_5_ (metasilicate), while vibrations of oxide β-Bi_2_O_3_ are not observed.

The longer-wavelength shoulder reappearing in the UV-visible spectra in Figure 7b around 450–500 nm upon annealing at 600 and 800 °C corresponds to the absorption of a narrower-gap bismuth sillenite Bi_12_SiO_20_ (a direct-gap semiconductor with *E_g_*~2.48 eV) [49]. As for sample BSO_hν_8 (having a cream color, just like sample BSO_8), its UV-Vis spectrum in Figure 7b shifts again to the longer-wavelength region, with the edge of the band corresponding to the absorption of bismuth orthosilicate Bi_4_Si_3_O_12_, just like for the non-irradiated sample BSO_8. Similarly, the Raman spectrum of sample BSO_hν_8 closely repeats that of its non-irradiated counterpart (sample BSO_8), which corresponds to a mixture of sillenite and bismuth orthosilicate. The bands in the FTIR spectra of laser-irradiated samples annealed at 500–800 °C resemble those of their non-irradiated counterparts (see above).

Thus, the phase evolution observed for samples of the BSO_hν group heated at 600 to 800 °C was quite similar to that in their non-radiated counterparts. Consequently, the overall transformations experienced by samples BSO_hν can be schematically represented as: amorphous phase → Bi_2_SiO_5_ → Bi_12_SiO_20_ → Bi_4_(SiO_4_)_3_.

#### 3.1.3. Comparison with Other Works

As the Bi–Si–O system was studied before, and different approaches were undertaken, there were several reports on how forming phases evolve in the system during thermal treatment. Using the sol–gel method, Dimitriev et al. observed amorphous material below 200 °C [26]. As temperature increased from 200 to 400 °C, this led to a gradual formation of Bi_4_Si_3_O_12_ which then crystallized to phase Bi_2_SiO_5_ at 600–800 °C. Silicate phases were observed to separate from the amorphous matrix upon heating [26]. Sakamoto with co-authors also calcined the product of the sol–gel synthesis and reported on β-Bi_2_O_3_ formation after 400 °C [3]. Afterwards, peaks of metasilicate Bi_2_SiO_5_ first appeared at 450 °C, with a single-phase sample forming at 500 °C. When the obtained material was then sintered (pressure-assisted sintering) to prepare a ceramic, phase Bi_2_SiO_5_ was detected up to 600 °C, then gradually turning to Bi_4_Si_3_O_12_ at 620 °C and finally to Bi_12_SiO_20_ at 640 °C [3]. Ke and co-authors used the solution deposition method to prepare thin bismuth-based films on substrate [2]. Above 400 °C, bismuth oxide (δ-modification) was observed to crystallize first from the amorphous matrix, after which its crystallinity increased at 450 °C, resulting in phase Bi_2_SiO_5_ emerging at 500 °C. Thus, even though the sequence of phase formation reported for bismuth silicates by others may differ, depending on thermal treatment and other conditions, formation of phase Bi_2_SiO_5_ at around 500 °C observed in the present work was previously mentioned in the literature.

Taniguchi with colleagues prepared Bi–Si–O glasses and described the evolution of phase composition which was quite similar to what was observed in the present work [25]. They heated samples from 300 to 700 °C, aiming at preparing Bi–Si–O glass from melt. Initially, amorphous XRD patterns with a maximum around 28° were recorded, similar to those seen for the BSO_hν group samples annealed at low temperatures. This implies that the homogeneity of elemental distribution in sample BSO_hν was quite close to that in the melt. The first well-pronounced crystalline peaks of Bi_2_O_3_ were detected at 470 °C in the work of Taniguchi et al. [25], also implying that bismuth oxide precipitated from an amorphous matrix. Then after 540 °C, XRD patterns of bismuth oxide and metasilicate began overlapping, after which phase Bi_2_SiO_5_ completely crystallized at 600 °C and then was observed at least up to 700 °C [25]. Note that the phase evolution observed in the present work was very similar, even though it was studied for nanomaterials, both non-irradiated and laser-irradiated before further heat treatment.

#### 3.1.4. Conclusions to Section 3.1

Thus, even though samples BSO and BSO_hν obtained via laser ablation and mixing of colloids (without or with further laser post-treatment) were inhomogeneous in terms of their local Bi/Si ratio, after annealing at different temperatures up to 800 °C they eventually resulted in bismuth silicate-based materials. Such nanomaterials are difficult to prepare via conventional approaches, which is why both the preparation technique and materials described in this study can find applications in some fields, especially after thorough characterization and optimization. The most important points that can be mentioned at this stage are as follows:

- Nanomaterials based on the pure bismuth metasilicate phase can be obtained through quite a simple synthetic approach.
- Nanomaterials based on mixtures of bismuth silicates can also be prepared by combining laser ablation and post-annealing at different temperatures, if necessary. Such mixed nanomaterials can exhibit properties comparable or superior to those based on pure metasilicate. Therefore, various nanocomposites obtained in this work are also of potential interest.
- At lower annealing temperatures (up to 500 °C) phase evolution of materials is governed very much by their initial state. That is, the products of annealing for non-irradiated and laser-irradiated samples BSO and BSO_hν differed significantly.
- The preparation technique described in this work, i.e., laser ablation in liquid (LAL) phase, is attractive for several reasons. It is not complex and can be combined with further annealing post-treatment, uses simple and inexpensive precursors, and is environmentally friendly. At the same time, it allows for a wide range of nanomaterials based on different bismuth silicates and their mixtures and, hence, with different properties.

### 3.2. Thermal Structure Evolution in Laser-Prepared Bismuth-Silicate-Based Nanomaterials

Along with phase evolution, the microstructure of annealed materials also changed during heating. To follow such changes, we chose the samples annealed at 300, 400, 600, and 800 °C to examine them by TEM and compared them with corresponding initial materials. This allowed us to reveal changes occurring with temperature and to compare the behavior of irradiated and non-irradiated materials.

#### 3.2.1. Samples of the BSO Group

The initial material BSO was described as agglomerates of nearly spherical particles in the range of 2.5–60 nm, with most of them being ~10 nm [20]. The BET specific surface area of this material was found to be of 40.1 cm^3^/g. Having locally, in general, an amorphous-like SAED pattern, it demonstrated the presence of crystalline Bi in its XRD pattern and Bi and Si in its Raman spectrum. 

**Figure 9:**
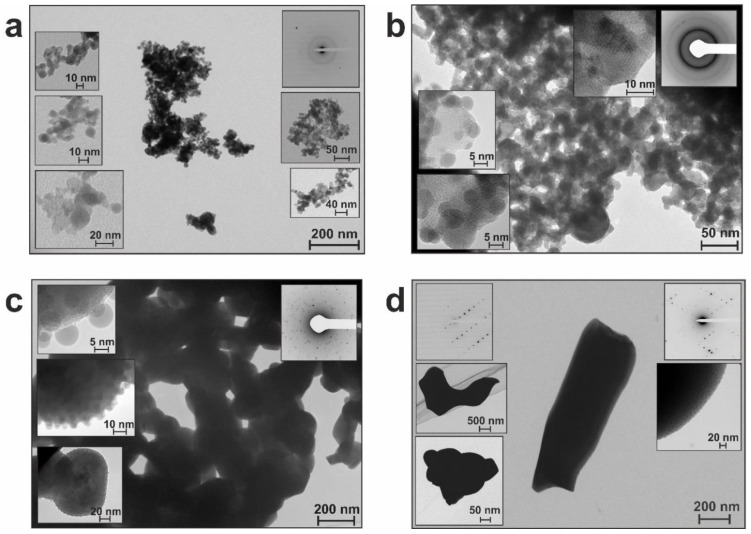
TEM images of selected samples from the BSO group: (**a**) BSO_3, (**b**) BSO_4, (**c**) BSO_6, and (**d**) BSO_8 annealed at 300, 400, 600 and 800 °C, respectively.

After heating at 300 °C, despite some crystal reflexes emerging in the SAED pattern (Figure 9a, inset), the general appearance of the material did not change (Figure 9a). However, annealing at 400 °C led to an increase in particle size to 3–80 nm (with the majority being ~12 nm) and decrease in *S*_BET_ to 27.2 cm^3^/g. Moreover, at this stage particles were found to begin segregating by sizes, with larger and rather shapeless ones (of 10–80 nm) tending to get covered with smaller ones (of 3–7 nm) (Figure 9b). This tendency was observed at 600 °C too, with smaller particles (3–10 nm) covering larger ones (20–300 nm), as clearly seen in Figure 9c. For sample BSO_6, as all particles predictably increased in size, their average values shifted to 80–100 nm, and *S*_BET_ became only 2.1 cm^3^/g, while their crystallinity was enhanced (see SAED pattern as inset in Figure 9c).

The highest crystallinity was demonstrated by the sample calcinated at 800 °C (see insets with SAED pattern in Figure 9d). In this sample, its larger particles grew as big as 200–500 nm (or even up to 1 µm), whereas its smaller particles remained basically unchanged (3–10 nm), still covering the larger ones quite densely. 

As discussed above, the phase composition of larger particles was revealed by XRD (see previous sections), whereas the smaller particles are believed to be amorphous silica whose crystallinity cannot be detected by XRD. In the Raman shift spectra, the peaks around 930 and 947 cm^–1^, which appeared for sample BSO_6, were ascribed to the stretching vibration of Si–O bonds [28] (see Section 3.1). Therefore, they might well belong to fine SiO_2_ particles located on the surface of bigger particles with larger crystallinity. The very first appearance of the two Raman peaks at 930 and 947 cm^−1^ was detected in the spectrum of sample BSO_5. They were still present in sample BSO_7, but could not be resolved in sample BSO_8, possibly because of overlap with other high-intensity bands in this sample.

In the FTIR spectra of samples annealed at 400 °C and above, the weak band at 675 cm^–1^ was attributed to bending vibrations of the Si–O bond [28,38] (see Section 3.1), which also supports the idea of small SiO_2_ particles on the surface of such heat-treated non-irradiated materials.

#### 3.2.2. Samples of the BSO_hν Group

**Figure 10:**
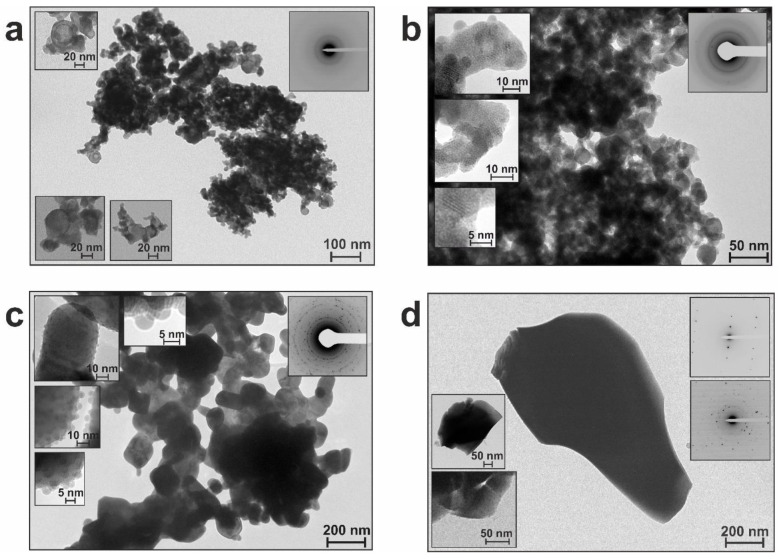
TEM images of selected samples from the BSO_hν group: (**a**) BSO_hν_3, (**b**) BSO_hν_4, (**c**) BSO_hν_6, and (**d**) BSO_hν_8 annealed at 300, 400, 600 and 800 °C, respectively.

The initial material BSO_hν was revealed to consist of agglomerates of rather shapeless amorphous particles [20]. Their size was estimated to be in the range of 3–50 nm, with most particles being ~10 nm. The *S*_BET_ of the sample was found to be of 56.9 cm^3^/g, which is larger than for its non-irradiated counterpart. Thermal treatment at 300 °C was found not to cause any visual changes in the sample’s appearance (Figure 10a), although the particle size distribution shifted to the range of 5–80 nm, with majority being around 10–12 nm in size. Nonetheless, the SAED pattern still remained unchanged, manifesting an amorphous material.

Heating at 400 °C resulted in a decrease in *S*_BET_ (28.9 cm^3^/g) and, similar to what was previously described for the non-irradiated sample BSO_4, particle segregation also began in the laser-irradiated sample BSO_hν_4. As clearly seen in Figure 10b, the larger particles (15–80 nm, most being around 15–20 nm in size) are covered with smaller ones (of 5–8 nm). Expectedly, the crystallinity of the larger particles somewhat increased at this point, as shown by the inset in Figure 10b.

As seen in Figure 10c, sample BSO_hν_6 can be described as consisting of larger particles (between 15 and 280 nm, with the majority being of 60–80 nm) covered with smaller spheres of 5–8 nm. Its specific surface area drastically dropped to 3.4 cm^3^/g, and its SAED pattern is typical of polycrystalline material (Figure 10c, inset).

Calcination at 800 °C led to a powder which looked very similar to non-irradiated sample BSO_8 annealed at the same temperature. As seen in Figure 10d, morphologically the two samples treated at 800 °C were also similar: larger particles of 0.2–1 µm formed in sample BSO_hν_8 were covered with smaller ones of 5–8 nm.

Since both the Raman and FTIR spectra of the BSO_hν group samples demonstrated the same evidence of SiO_2_ presence (as was above discussed for the BSO series), we concluded that the smaller particles were also silica. As for the larger particles, their phase evolution was described and discussed along with XRD results in the previous section.

#### 3.2.3. Discussion to Section 3.2

As expected, temperature treatment of both samples was found to lead to both increased size and crystallinity of their particles (Figure S3, Supplementary Materials). Naturally, the specific surface area of the powders decreased.

Initially, the materials could be described either as a mechanical mixture (BSO) of ablated Bi and SiO_x_/Si species or as a similar mixture somehow “fused” by laser irradiation (BSO_hν). Then, as the samples were annealed at gradually elevated temperatures, further interaction of their Bi and Si components occurred (see previous sections). As a result, not only the formation of Bi–Si–O phases but also significant morphological changes (i.e, phase and particle segregation) were found to occur. More precisely, the samples segregated into larger particles with better crystallinity (mainly based on Bi–O and Bi–Si–O phases) and smaller ones located on their surface (based on amorphous silica). The number of the latter smaller particles was found to grow gradually with annealing temperature, while their size and shape remained mainly unchanged.

In general, appearance of smaller particles on larger ones was previously reported by others. A similar segregation of particles by sizes in Bi-based systems was also observed by Liu et al. (Bi_4_O_5_Br_2_/Bi_24_O_31_Br_10_ nanosheets covered with Bi_2_SiO_5_ particles) [50], by Naing et al. (Bi particles on the surface of montmorillonite-loaded BiOCl) [51], and by Huang et al. (Bi nanoparticles on Bi_2_WO_6_) [52], to name a few. Most such products were obtained through the hydrothermal (or solvothermal) synthesis, in which case mixing, i.e., homogenization, of the reaction mixture was possible. The resulting product consisted of two (or more) types of particles, and thus the distribution of smaller particles over the surface of larger ones could indicate the extent of homogeneity of the entire product.

Note that the composition of smaller particles mentioned above was different in different works, from crystalline Si, to metallic Bi, or to Bi_2_SiO_5_. In this study, we assumed the observed small particles were based on SiO_2_. Since they remained amorphous, such small particles could not be well probed by XRD, and only surface analysis methods (such as Raman and FTIR spectroscopy) supported their nature as being SiO_2_. Moreover, we hypothesized the following genesis of the smaller particles. Both initial samples, namely BSO and BSO_hν, were presented with quite small particles of 2.5–60 and 3–50 nm, respectively. As annealing temperature was elevated, some of the particles began growing bigger, while others remained mainly unchanged. And as the larger particles were found to grow with temperature, the difference between the two types increased, thus revealing that the smaller particles, which were found in all the samples, including those calcinated at 800 °C, were present in the materials from the very beginning. They were hard to distinguish in non-annealed samples (see Figure S4, Supplementary Material), but became more visible in annealed materials (against the background of large crystalline particles), as their shape and size did not change much with temperature. Since the number of such smaller particles appeared to be larger after annealing, we assume that as the bigger particles (with BSO-based phases) crystallized better, they tended gradually to squeeze out the silica phase onto their surface. This explains well why the maximum concentration of silica particles was observed on the surface of both samples annealed at 800 °C. Therefore, the smaller particles seen in all the samples are silica present in the initial materials that did not change much under temperature treatment. Consequently, the obtained materials seem to be SiO_2_/Bi–Si–O composites with different Bi-containing phases depending on annealing temperature. It should also be noted that such fine SiO_2_ particles present in all the samples could be partially a source of Si for any thermal phase transformation.

In any case, in this study, the segregation of silica particles on the surface of another phase cannot necessarily be considered as an undesirable or interfering process. Firstly, their uniform distribution indicates a high homogeneity of the resulting composite material. Secondly, the resultant composite material can find a lot of applications specifically due to the observed combination with silica. Its presence can modify and improve the properties of a composite material, being its component (see, for example [53,54]). Moreover, composites with silicon dioxide were reported to be used in a number of applications, e.g., as antibacterial agents [55], for wastewater treatment [56], for liquid chromatography [57], to enhance the microstructure and mechanical performance of cement composites [58], etc.

#### 3.2.4. Conclusions to Section 3.2

In principle, the growth of particle size and crystallinity, as well the reduction of *S*_BET_, are quite expected results of thermal evolution in nanomaterials. At the same time, the segregation and assembly of fine SiO_2_ particles (3–10 nm in size) on larger bismuth-silicate particles largely observed above 400 °C is a new finding of this study. Eventually, since the fine particles are distributed over their larger ones rather uniformly, the materials obtained at higher annealing temperatures are expected to be quite homogeneous. Based on their composition (revealed in this study), they should be treated as composites of bismuth silicate(s) and SiO_2_, with somewhat tunable compositions that can find their applications in different fields.

### 3.3. Photocatalytic Activity of the Laser-Prepared Bismuth-Silicate-Based Nanomaterials

**Figure 11:**
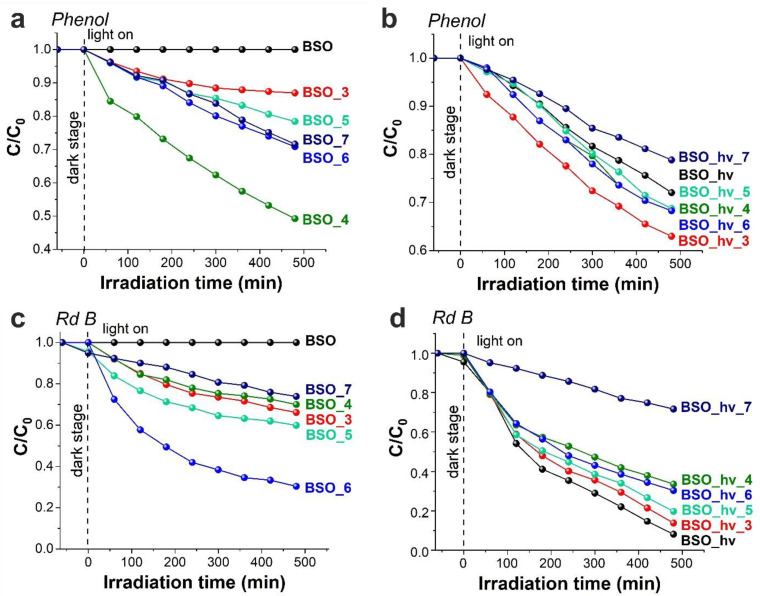
Kinetic curves for phenol (**a**,**b**) and Rd B (**c**,**d**) decomposition in presence of BSO (**a**,**c**) and BSO_hν (**b**,**d**) groups of samples.

The photocatalytic efficiency of materials towards decomposition of Rd B was assessed by means of absorption spectra (Figure S5a, Supplementary Material), while phenol decay was studied by fluorescence spectroscopy, since the absorption peaks of photoproducts formed during its decomposition are known to overlap with those of phenol (Figure S5b, inset) [20,59]. The results of PCA studies of the BSO-based materials prepared in the present work are presented in Figure 11 and in Table 2.

#### 3.3.1. Photocatalytic Decomposition of Phenol

Under the chosen experimental conditions, the photodecomposition of phenol can be described as a first-order reaction (Figure 11a,b). Photodecomposition of phenol in the absence of a catalyst is not expected as it does not absorb at a wavelength of 375 nm (used for illumination). Also, the intermediate products formed during its photolysis are known practically not to absorb in this region [60].

The initial non-radiated sample BSO, which was found to have some metallic Bi and amorphous silicon/silica phases, did not decay phenol at all. After annealing at 200–500 °C (samples BSO_2– BSO_5), when bismuth β-oxide was observed to form in the samples, the materials immediately began to exhibit PCA against phenol. The best result was demonstrated by sample BSO_4 (its decomposition rate constant *K_app_* being 3.9 µM/h) which had the maximal content of phase β-Bi_2_O_3_ in the BSO group samples. According to the literature, it is the β-oxide that exhibits the highest PCA among all bismuth oxides [61,62]. A small amount of Bi_2_SiO_5_ found in this sample, apparently, did not affect its PCA.

A significant increase in the content of bismuth metasilicate phase in sample BSO_5 was found to result in a sharp drop in its PCA. Using the approach previously published in work [63], we constructed energy diagrams for the systems containing beta-oxide and bismuth silicates (see Figure S6, Supplementary Material). They show that for the coupled system of phases β-Bi_2_O_3_ and Bi_2_SiO_5_, it is possible to form a heterostructure of type I which does not lead to an increase in its PCA. According to work [64], such a heterostructure of *n*-type (Bi_2_SiO_5_) and *p*-type (β-Bi_2_O_3_) semiconductors can transform into type II upon photoexcitation, which causes an increase in PCA. However, the authors of work [64] observed small clusters of β-Bi_2_O_3_ formed on the surface of larger Bi_2_SiO_5_ particles, while in the present work, β-oxide was found to form from metal particles and comprised the main phase, whereas the formation of bismuth metasilicate occurred afterwards, during the decomposition of β-oxide in presence of Si (or Si oxide) clusters. So, morphologically and chemically, our composite materials were completely different, implying that a similar transformation of their heterostructure type was not likely to occur. 

With a further increase in annealing temperature, sample BSO_6, which was found to contain essentially a single phase of bismuth metasilicate, demonstrated enhanced PCA in comparison with material BSO_5 (Figure S7). The latter activity remained almost unchanged for sample BSO_7 heat-treated at 700 °C, despite the appearance of bismuth orthosilicate and sillenite phases in the sample. After annealing at 800 °C, when the sample was found to contain primarily the orthosilicate phase, no photocatalytic activity was observed.

The highest PCA towards phenol decomposition observed in the BSO_hν group was demonstrated by amorphous samples (Figure 11). Among them, the maximal efficiency was achieved by sample BSO_hν_3 (*K_app_* = 3.0 µM/h, Figure S7), whose material very likely contained β-Bi_2_O_3_ according to XRD data (Figure S2). The formation of ordered Bi_2_SiO_5_ crystal structure in samples BSO_hν_5 and BSO_hν_6, along with a simultaneous decrease in specific surface area of the samples, led to a slight decrease in their PCA (*K_app_* = 2.7 µM/h, Figure S7). Then a further increase in annealing temperature was found to result in the formation of bismuth orthosilicate as a primary phase and disappearance of photo-activity in sample BSO_hν_8.

#### 3.3.2. Photocatalytic Decomposition of Rd B

Unlike phenol, Rhodamine B absorbs the used excitation radiation, and so do the tested photocatalysts. Therefore, photo-processes can proceed at both the ground and excited states of this dye, wherein the decomposition of Rd B can follow different mechanisms, e.g., immediately with the destruction of the aromatic structure and without shifting the maximum of the absorption spectrum [65]. When the dye is irradiated in the presence of the studied nanomaterials, its decomposition occurs with a characteristic hypsochromic shift of the absorption maximum from 553 to 495 nm (Figure S5). According to reports [66,67], the hypsochromic shift is associated with the formation of a number of N-deethylated Rd B intermediates, including Rhodamine 110, which has an absorption maximum at 495 nm. N-deethylation proceeds without decomposition of the aromatic structure of the dye. Considering that N-diethylated products practically do not absorb in the region of the absorption maximum of Rh B, in this work we determined the constants of N-diethylation (*K*_N_) from the decrease in optical density at a wavelength of 553 nm. In addition, since the optical densities of Rd B and Rhodamine 110 are close for same concentrations, we also estimated the photodecomposition rate constant of the aromatic structure of the dye (*K_app_*). It was calculated from kinetic curves, according to the maximal optical density in the region of 400–553 nm. Based on the obtained results, we assumed that the process of N-diethylation occurs an order of magnitude faster than the process of degradation of the aromatic structure, and the increase in the rate of N-diethylation does not always correlate with the rate of Rd B degradation. Therefore, below we focus in more detail on the photocatalytic decomposition of the aromatic structure of the dye.

In the BSO group, similar to the case of phenol, the initial non-irradiated sample BSO was found not to exhibit PCA towards Rd B. However, samples containing phase β-Bi_2_O_3_ showed relatively low activity towards Rd B (in contrast to their activity towards phenol), so that for sample BSO_3 its *K_app_* was only 0.2 µM/h. Formation of the bismuth metasilicate phase was observed to increase the rate of Rd B decomposition. For sample BSO_6 containing 97% of Bi_2_SiO_5_, the rate constant increases to 0.6 µM/h. Finally, sample BSO_8 containing bismuth orthosilicate did not exhibit PCA towards either Rd B or phenol.

In the BSO_hν group, samples with bismuth metasilicate (Bi_2_SiO_5_) exhibited good PCA. At the same time, the non-annealed sample BSO_hν (*K_app_* = 1.5 µM/h) exhibited the best result, having decomposed ~95% of the aromatic structure of the dye after 8 h of irradiation (see Figure 11d, black curve). The increase in calcination temperature, which leads to a decrease in the specific surface area and formation of crystalline bismuth metasilicate, was found to result in lower photodecomposition rates of Rd B. Finally, sample BSO_hν_8 containing primarily bismuth orthosilicate exhibited no PCA, similar to its inactivity towards phenol.

#### 3.3.3. Discussion and Conclusions to Section 3.3

Silicates and other complex oxides based on bismuth (such as vanadates, titanates and so on) are attractive as photocatalysts for the decomposition of various organic compounds. This is well seen in Table 3, which compares photocatalytic performance of such nanomaterials recently reported in the literature with those presented in this study.

According to the energy diagram in Figure S6, for almost all variants of phase composition in the samples studied in this work, the formation of type I heterostructures is possible. Thus, one can assume a predominantly independent action of available phases in photocatalytic processes. Basically, this is what was observed in this work, which is why at this point one can somehow hypothesize about the PCA of different phases. As for the difference in specific surface area of the powders and its influence on the PCA results obtained, Figure S8 shows that no convincing correlation between *S*_BET_ and *K_app_* can be found.

Phenol degradation was found to proceed better in the presence of samples containing crystalline or amorphous β-Bi_2_O_3_ (Figure S2a,b). Then, slightly lower PCA towards phenol decomposition was exhibited by samples based on Bi_2_SiO_5_. As for Rd B degradation, metasilicate-based materials from both BSO and BSO_hν groups exhibited high activity (about 70–80% degradation after 8 h). However, the best photocatalytic performance was demonstrated by the initial amorphous BSO_hν material and sample BSO_hν_3 annealed at 300 °C. Since β-Bi_2_O_3_ was concluded to be not very active towards Rd B degradation in the BSO group samples, we assume some other phase as the active component in sample BSO_hν_3.

In general, according to the data presented in Figure S7 (Supplementary Material), the laser-irradiated (or BSO_hν group) materials tended to exhibit better photocatalytic performance than their non-irradiated counterparts from the BSO group.

## 4. Conclusions

The present work focused on careful analysis of structure and phase evolution of two samples in the Bi–Si–O system (with the initial Bi/Si ratio of 2:1) during their annealing up to 800 °C, as nanomaterials based on bismuth silicates are difficult to prepare but are of potentially high interest for various applications. Both initial samples were based on laser-generated Bi and Si colloids prepared in water and then mixed, while one of them was additionally irradiated in water by the same pulsed laser as a post-treatment stage. The laser post-treatment was observed to cause significant changes in initial samples and influenced their phase transformations at temperatures approximately below 500 °C. At the same time, after annealing at higher temperatures, the difference between laser-irradiated and non-irradiated samples tended to be less noticeable.

In general, the phase evolution after 500 °C goes with increasing symmetry, from Bi_2_SiO_5_ to Bi_12_SiO_20_, and then to Bi_4_(SiO_4_)_3_. It should be highlighted that using the method described here, pure metasilicate phase can be obtained under certain conditions, which is quite a significant result. 

During annealing, quite expectedly, growth of particle size and crystallinity, as well as reduction of specific surface area, were observed along with the formation of different Bi oxide and silicate phases and their transformations. Also, segregation and assembly of fine SiO_2_ particles (3–10 nm in size) around larger bismuth-silicate particles was observed at temperatures > 400 °C, implying that most of the prepared samples were SiO_2_/Bi–Si–O composites with different Bi-containing phases depending on annealing temperature.

Photocatalytic activity of all obtained powders was studied in terms of their decay of phenol and Rhodamine B. Phenol degradation was found to proceed better in the presence of samples containing crystalline or amorphous β-Bi_2_O_3_, after which followed samples based on Bi_2_SiO_5_. For Rhodamine B degradation, metasilicate-based materials exhibited high activity, while the best photocatalytic performance was demonstrated by the initial amorphous sample post-irradiated by laser. In general, laser-irradiated materials tended to exhibit better photocatalytic performance than their non-irradiated counterparts.

The preparation approach used in this work (laser ablation with further laser post-treatment in water followed by subsequent temperature control of the structure and properties of produced nanomaterials) proved to be promising. It can be used for the synthesis of bismuth silicates of various stoichiometries, as well as for other catalytically active materials based on complex oxides of Bi (e.g., in the Bi-Ti-O and Bi-V-O systems) and their composites.

## Figures and Tables

**Table 1:** *E_g_* values (eV) for BSO and BSO_hν group samples obtained from DRS data.

Sample	Tauc Method, Type of Transition		DASF Method
	Direct Permitted (*n* = 1/2)	Indirect Permitted (*n* = 2)	
BSO	**-**	**-**	**-**
BSO_2	**-**	**-**	**-**
BSO_3	3.1 (Bi_2_SiO_5_)	2.3 (β-Bi_2_O_3_)	2.5 (β-Bi_2_O_3_)/3.4 (Bi_2_SiO_5_)
BSO_4	3.2 (Bi_2_SiO_5_)	2.3 (β-Bi_2_O_3_)	2.5 (β-Bi_2_O_3_)/3.5 (Bi_2_SiO_5_)
BSO_5	3.6 (Bi_2_SiO_5_)	2.2 (β-Bi_2_O_3_)	2.4 (β-Bi_2_O_3_)/3.8 (Bi_2_SiO_5_)
BSO_6	3.7 (Bi_2_SiO_5_)	-	-/3.8 (Bi_2_SiO_5_)
BSO_8	2.3 (Bi_12_SiO_20_)	3.2 (Bi_4_Si_3_O_12_)	2.3 (Bi_12_SiO_20_)/3.4 (Bi_4_Si_3_O_12_)
BSO_hν	3.6 (Bi_2_SiO_5_)	-	-/3.6 (Bi_2_SiO_5_)
BSO_hν_2	3.5 (Bi_2_SiO_5_)	-	-/3.5 (Bi_2_SiO_5_)
BSO_hν_3	3.5 (Bi_2_SiO_5_)	-	-/3.4 (Bi_2_SiO_5_)
BSO_hν_4	3.5 (Bi_2_SiO_5_)	-	-/3.3 (Bi_2_SiO_5_)
BSO_hν_5	3.7 (Bi_2_SiO_5_)	-	-/3.6 (Bi_2_SiO_5_)
BSO_hν_6	3.7 (Bi_2_SiO_5_)	-	-/3.7 (Bi_2_SiO_5_)
BSO_hν_8	2.3 (Bi_12_SiO_20_)	3.1 (Bi_4_Si_3_O_12_)	2.3 (Bi_12_SiO_20_)/3.3 (Bi_4_Si_3_O_12_)

**Table 2:** Photodecomposition rate constants for phenol and Rd B in μM/h.

Sample	Phenol *K_app_*	Rd B		Sample	Phenol *K_app_*	Rd B	
		*K*_N_	*K*_app_			*K*_N_	*K*_app_
BSO	–	–	–	BSO_hν	2.1	4.8	1.5
BSO_3	0.6	0.6	0.2	BSO_hν_3	3.0	2.2	1.1
BSO_4	3.9	0.7	0.2	BSO_hν_4	2.7	9.3	0.6
BSO_5	1.2	3.1	0.3	BSO_hν_5	2.7	2.8	0.8
BSO_6	2.1	3.1	0.6	BSO_hν_6	2.7	1.0	0.6
BSO_7	2.1	0.5	0.2	BSO_hν_7	1.5	4.8	0.2
BSO_8	–	<0.1	–	BSO_hν_8	–	<0.1	–

**Table 3:** Photocatalytic efficiency of complex oxides based on bismuth.

Photocatalysts	Preparation Method	Photocatalytic Experiment Conditions	Photocatalytic Efficiency	Ref.
Bi_2_SiO_5_ and Bi_4_(SiO_4_)_3_ nanofibers	Electrospinning technique	250 W high-pressure mercury lamp (311 mW/cm^2^); 1.5 g catalyst; methyl orange (MO), 12.5 mmol/L; safranin O (SO), 12.5 mmol/L	Max. *k_app_* for Bi_4_(SiO_4_)_3_: 0.273 min^–1^ (MO), 0.409 min^–1^ (SO); for Bi_2_SiO_5_ 0.149 min^–1^ (MO), 0.301 min^–1^ (SO)	[15]
Bi_2_SiO_5_, Bi_12_SiO_20_, Bi_4_Si_3_O_12_	Controlled hydrothermal method	15 W Xe lamp (0.312 W/cm^2^); 0.1 mg/mL catalyst; crystal violet (CV), 10 ppm	Max *k_app_* for Bi_2_SiO_5_: 2.57 × 10^−2^ h^–1^	[18]
Bi_2_SiO_5_/BiOBr type-II heterojunction	In-situ partial ion exchange strategy	300 W Xe lamp; 0.6 mg/mL catalyst; RhB, 2.0 × 10^−5^ mol L^−1^	Max *k_app_* for sample Bi_2_SiO_5_/BiOBr-90: 0.07522 min^–1^	[28]
OVs-Bi_2_O_3_/Bi_2_SiO_5_ microsphere heterojunctions	One-pot solvothermal synthesis	500 W Xe lamp; 1 mg/mL catalyst; MO, 10 mg/L; phenol (PhOH), 10 mg/L	Max *k_app_* for sample (1.5%) Bi_2_O_3_/Bi_2_SiO_5_: 0.159 h^–1^ (MO), 0.059 h^–1^ (PhOH)	[30]
The flower-like Pt/Bi_2_SiO_5_	Hydrothermal-photoreduction method	20 W Hg lamp; 0.5 mg/mL catalyst; 17α-ethynylestradiol (EE2), 3 mg/L	Max *k_app_* for 0.2Pt/BSO: 0.3629 min^−1^	[32]
Bi_4_(SiO_4_)_3_/Bi_2_SiO_5_ nanosheet	One-pot hydrothermal process.	300 W Xe lamp; 1 mg/mL catalyst; RhB, 10 mg L^−1^; PhOH, 20 mg L^−1^	Max *k_app_* for BSO-HNS: 0.064 min^−1^ (RhB), and 35% decomposition of PhOH per 150 min irradiation	[43]
Bi_4_O_5_Br_2_/Bi_24_O_31_Br_10_/Bi_2_SiO_5_ heterostructure	In situ ion exchange reaction	500 W Xe lamp (38 mW cm^−2^); 0.6 mg/mL catalyst; PhOH, 5 mg/L	Max *k_app_* for Bi_4_O_5_Br_2_/Bi_24_O_31_Br_10_/Bi_2_SiO_5_ (S1): 0.07 h^–1^	[50]
Bi_2_O_3_/Bi_2_SiO_5_ p-n heterojunction	One-step calcination method from Bi(NO_3_)_3_ and SiO_2_	500 W Xe lamp (35 mW/cm^2^); 2 mg/mL catalyst; methylene blue (MB), 100 ppm; PhOH, 10 ppm; 2,4-dichlorophenol (2,4-DCP), 10 ppm	Max *k_app_* for Bi_2_O_3_/Bi_2_SiO_5_ (Bi/Si-4): 0.26 h^–1^ (MB), 0.2 h^–1^ (PhOH), 1 h^−1^ (2,4-DCP)	[64]
3D Bi_2_SiO_5_ hierarchical microspheres	Solvothermal method	300 W Hg lamp; 1 mg/mL catalyst; RhB, 10 mg/L; PhOH, 20 mg/L	Decolorization of RhB 90% and decomposition of PhOH 44% per 30 min	[68]
Hybrid Bi_2_SiO_5_ mesoporous microspheres	Hydrothermal method with “Postsynthetic modification”	500 W Xe lamp; 1 g/L catalyst; tetraethylated RhB, 1 × 10^−5^ M	Degradation of RhB 80% per 2 h for mesoporous Bi_2_SiO_5_	[69]
Bi_2_SiO_5_/BiPO_4_ heterostructure	Co-precipitation hydrothermal method	500 W xenon lamp; 0.6 mg/mL catalyst; PhOH, 10 ppm; MB, 2 × 10^−5^ M	Max *k_app_*: 0.00946 min^−1^ (PhOH), 0.00953 min^−1^ (MB)	[70]
Layered Bi_2_SiO_5_ and body-centered Bi_12_SiO_20_	Hydrothermal method	300 W Xe arc lamp (>420 nm and ≤420 nm); 0.5 mg/mL catalyst; rhodamine B (RhB), 10 mg/L	Max *k_app_*: 0.004 min^–1^ (>420 nm)	[71]
Bi_2_SiO_5_-Br, Bi_2_SiO_5_-Cl nanoparticles	Hydrothermal method	137 W Xe lamp; 1 mg/mL catalyst; RdB, 50 μM; tetracycline, 20 ppm	Max *k_app_* for Bi_2_SiO_5_-Br: 0.087 min^−1^ (RdB), Degradation tetracycline 83% per 180 min	[72]
Bi_2_SiO_5_ flower-like microsphere	Ion exchange method	100 W high pressure Hg lamp (λ ≈ 365 nm), 500 W Xe lamp (λ ≥ 420 nm); 0.6 mg/mL catalyst; PhOH, 5 ppm	Max *k_app_*: 1.6 h^–1^ (λ ≈ 365 nm), 0.4 h^–1^ (λ ≥ 420 nm)	[73]
Self-modified Bi_2_SiO_5_/Bi_12_SiO_20_ heterojunction	Citric acid-assisted hydrothermal method	100 W high pressure Hg lamp; 1 mg/mL catalyst; acid orange 7 (AO7), 20 mg L^−1^	Max *k_app_*: 0.1694 min^−1^	[74]
Mesoporous Bi_2_O_3_/Bi_2_SiO_5_@ SiO_2_ composite	Hydrothermal method	500 W Xe arc lamp; 0.5 g/L catalyst; bisphenol A (BPA), 20 mg L^−1^	Degradation BPA 90% per 120 min	[75]
Bi_2_SiO_5_/Bi_12_SiO_20_ heterojunction	Microwave hydrothermal synthesis	250 W Hg lamp; 0.05 mg/mL catalyst; RhB) 10 mg/L; MB, 20 mg/L	Max *k_app_*: 0.095min^−1^ (RhB), 0.083 min^−1^ (MB)	[76]
Spherical-Shaped BiVO_4_	Hydrothermal method	300 W Xe lamp; 1 mg/mL catalyst; crystal violet, 0.5 mM	Max *k_app_*: 5.88 × 10^−6^ s^–1^	[77]
BiVO_4_	Hydrothermal method	250 W metal halide lamp; 2 mg/mL catalyst; MB, 10 mg/L	Max *k_app_*: 0.015 min^−1^	[78]
Nanostructures Bi_12_TiO_20_	Hydrothermal method	1000 W halogen lamp; 1 mg/mL catalyst; AO7, 20 mg L^−1^	Max *k_app_*: 0.327 h^–1^	[79]
β-Bi_2_O_3_/Bi_2_SiO_5_, Bi_2_SiO_5_, Bi_12_SiO_20_/Bi_4_Si_3_O_12_	LAL, powder annealing	51 mW LED (375 nm); 0.5 mg/mL catalyst; Rd B, 5 µM; PhOH, 50 µM	Max *K_app_* 3.9 μM/h Bi_2_O_3_/Bi_2_SiO_5_, PhOH); 1.5 μM/h (amorphous Bi_2_SiO_5_, Rd B)	This work

## Data Availability

The data presented in this study are available on request from the corresponding authors.

## Supplementary Materials

The following supporting information can be downloaded at: https://www.mdpi.com/article/10.3390/nano12224101/s1, Figure S1: An example of band gap estimation for sample BSO_4: (**a**) Tauc method, short-wavelength band, direct-gap transition of Bi_2_SiO_5_; (**b**) long-wavelength band, non-direct-gap transition of β-Bi_2_O_3_; (**c**) DASF method; Figure S2: XRD patterns of non-irradiated samples BSO (**a**) and laser-irradiated samples BSO_hν (**b**) before and after thermal treatment, with detected phases marked; Figure S3: Evolution of particle size distribution for samples BSO (**top row**) and BSO_hν (**bottom row**) at selected temperatures (initial → 400 °C → 600 °C); Figure S4: TEM images of initial samples BSO (**a**) and BSO_hν (**b**). Spherical silica-based particles are marked with red arrows; Figure S5: Changes in fluorescence spectra upon photodecomposition of phenol (**a**), and absorption spectra upon photodecomposition of Rd B (**b**). Inset in panel (**a**) shows absorption spectra of phenol; Figure S6: Energy diagram for β-Bi_2_O_3_ and bismuth silicates; Figure S7: Photodecomposition rate constants for (**a**) phenol and (**b**) Rd B versus annealing temperature of photocatalysts; Figure S8: Photodecomposition rate constants difference in comparison with *S*_BET_ difference for some samples from the BSO and BSO_hν groups; Table S1: Phase composition of samples obtained from XRD data.
