# Supplementary material for: Phase and Structural Thermal Evolution of Bi–Si–O Catalysts Obtained via Laser Ablation
Source: Nanomaterials (Basel). 2022 Nov 21;12(22):4101. doi: 10.3390/nano12224101 (PMC9694683; doi:10.3390/nano12224101)
Supplement: Supplementary file 1 [file nanomaterials-12-04101-s001.zip › nanomaterials-2029626-supplementary.pdf]

nanomaterials

Supplementary

Phase and Structural Thermal Evolution of Bi–Si-O Catalysts Obtained via Laser Ablation

Anastasiia V. Shabalina [1,*], Alexandra G. Golubovskaya [1], Elena D. Fakhrutdinova [1], Sergei A. Kulinich [2,*], Olga V. Vodyankina [3] and Valery A. Svetlichyi [1]

1 Laboratory of Advanced Materials and Technology, Tomsk State University, Tomsk 634050, Russia
2 Research Institute of Science & Technology, Tokai University, Hiratsuka 259-1292, Japan
3 Laboratory of Catalytic Research, Tomsk State University, Tomsk 634050, Russia
* Correspondence: shabalinaav-1985@yandex.ru (A.V.S.); skulinich@tokai-u.jp (S.A.K.)

Supplementary Materials

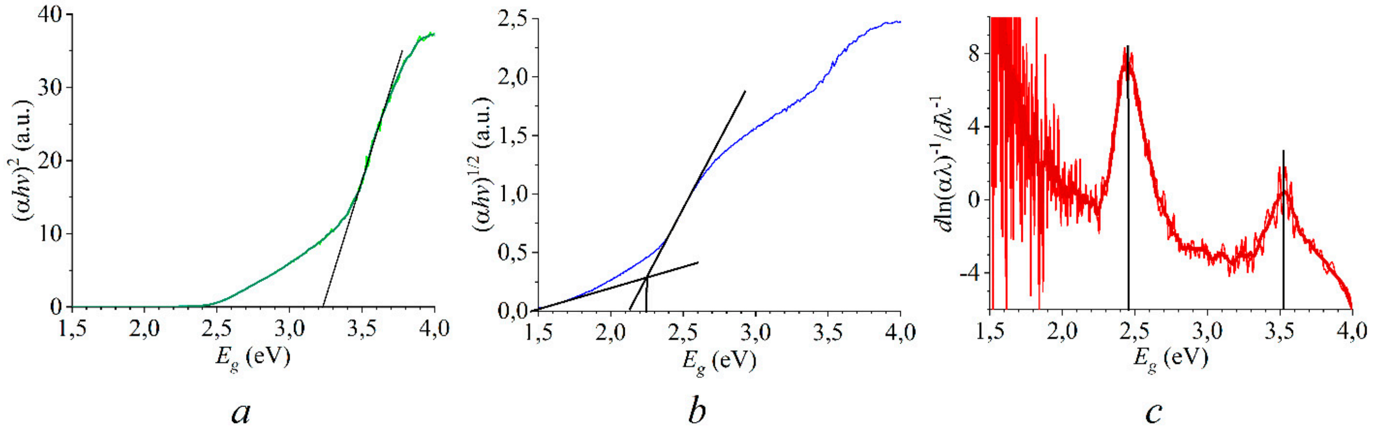

Figure S1. An example of band gap estimation for sample BSO_4: (a) Tauc method, short-wavelength band, direct-gap transition of Bi_2SiO_5; (b) long-wavelength band, non-direct-gap transition of β-Bi_2O_3; (c) DASF method.

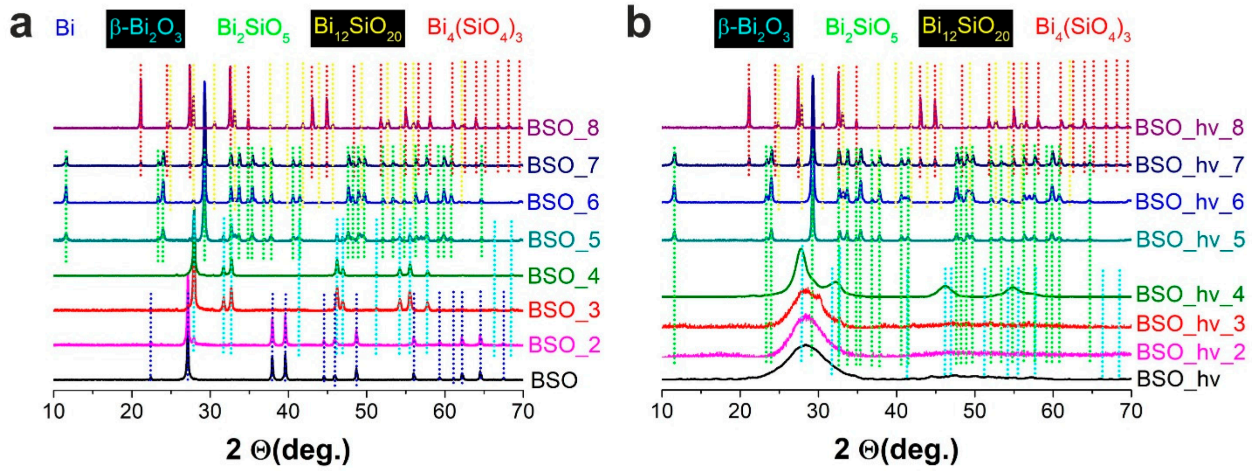

Figure S2. XRD patterns of non-irradiated samples BSO (a) and laser-irradiated samples BSO_hv (b) before and after thermal treatment, with detected phases marked.

Table S1. Phase composition of samples obtained from XRD data.

Sample	Phase content (%)			
	Bi	β-Bi_2O_3	Bi_2SiO_5	$Bi_{12}SiO_{20}$
BSO	100	–	–	–
BSO_2	70	30	–	–
BSO_3	3	97	–	–
BSO_4	–	100	–	–
BSO_5	–	15	85	–
BSO_6	–	–	97	3
BSO_7	–	–	90	4
BSO_8	–	–	–	30
BSO_hv	amorphous			
BSO_hv_2	amorphous			
BSO_hv_3	amorphous			
BSO_hv_4	amorphous			
BSO_hv_5	–	–	100	–
BSO_hv_6	–	–	98	2
BSO_hv_7	–	–	93	2
BSO_hv_8	–	–	–	30

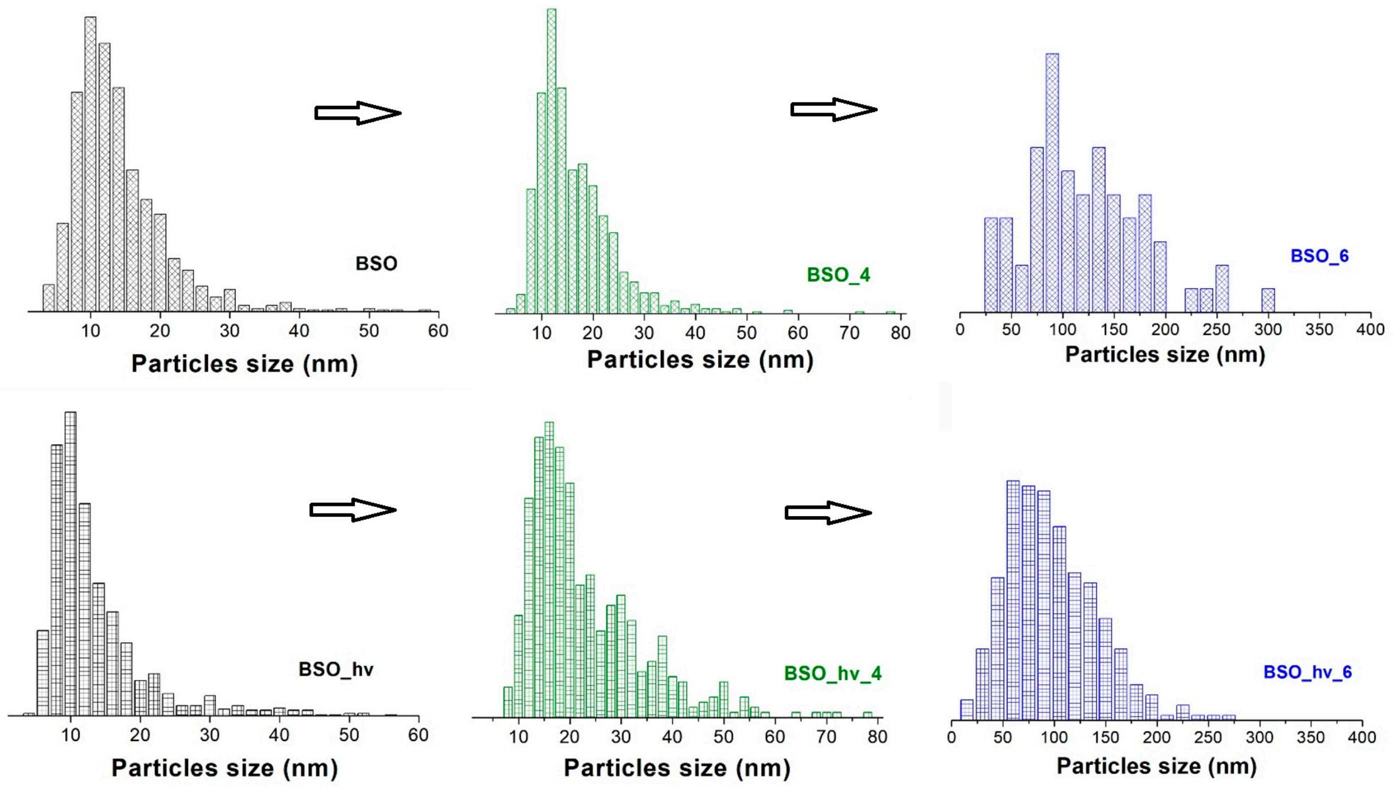

Figure S3. Evolution of particle size distribution for samples BSO (top row) and BSO_hv (bottom row) at selected temperatures (initial → 400 °C → 600 °C).

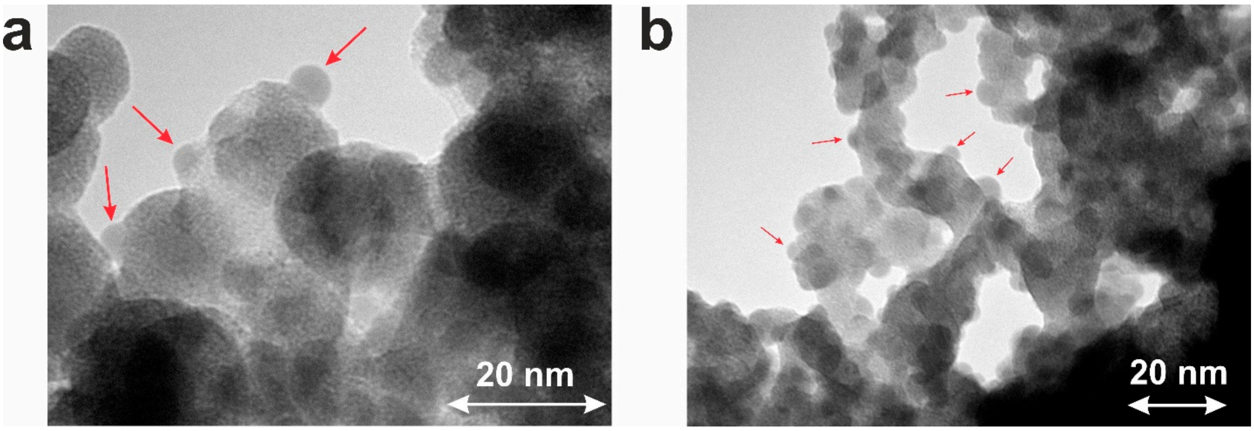

Figure S4. TEM images of initial samples BSO (a) and BSO_hv (b). Spherical silica-based particles are marked with red arrows.

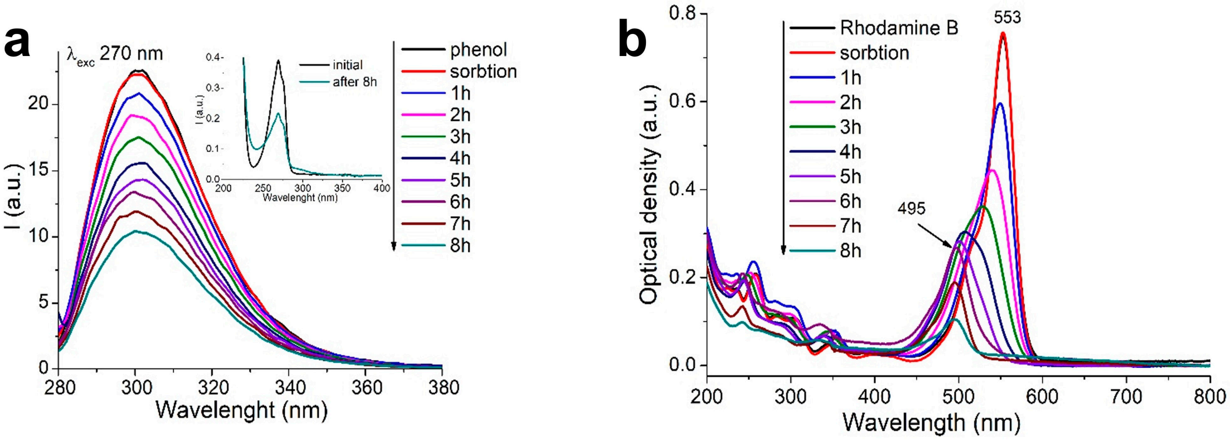

Figure S5. Changes in fluorescence spectra upon photodecomposition of phenol (a), and absorption spectra upon photodecomposition of Rd B (b). Inset in panel (a) shows absorption spectra of phenol.

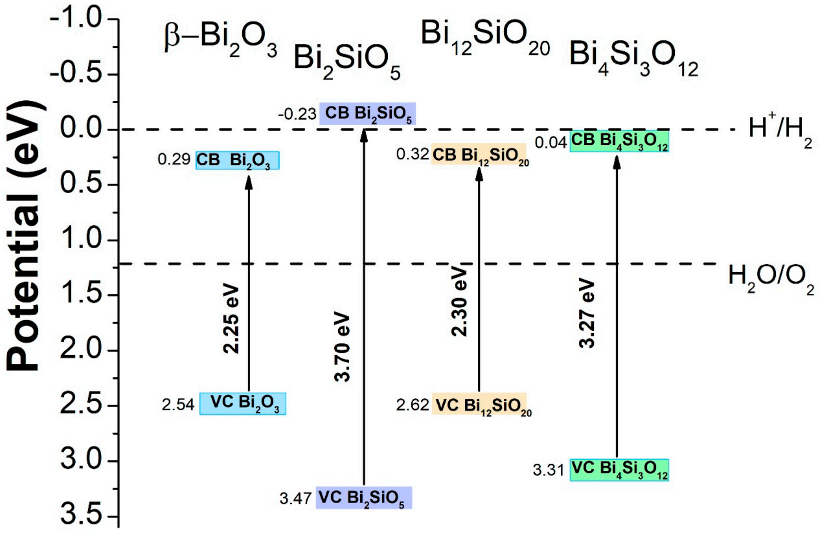

Figure S6. Energy diagram for β-Bi_2O_3 and bismuth silicates.

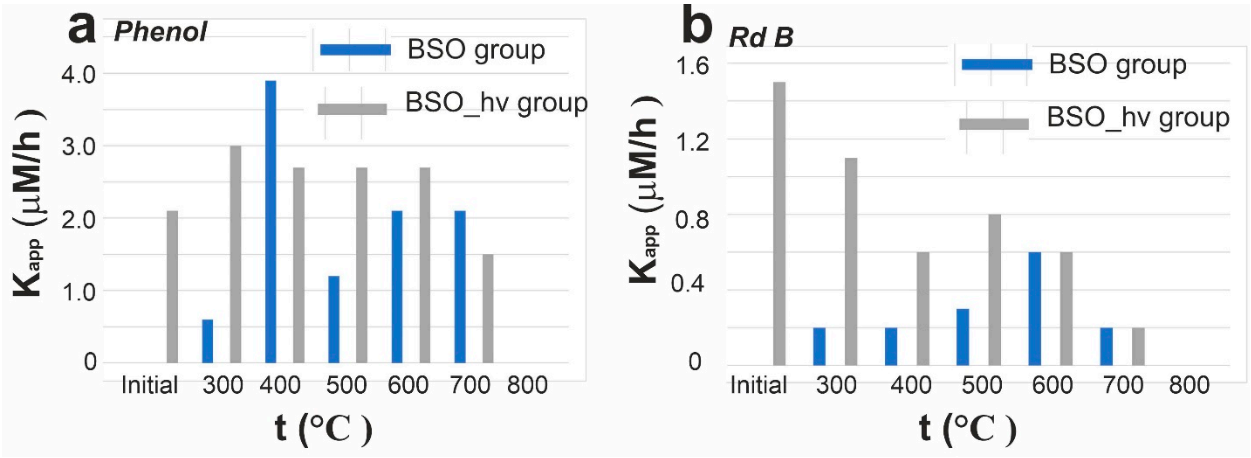

Figure S7. Photodecomposition rate constants for (a) phenol and (b) Rd B *versus* annealing temperature of photocatalysts.

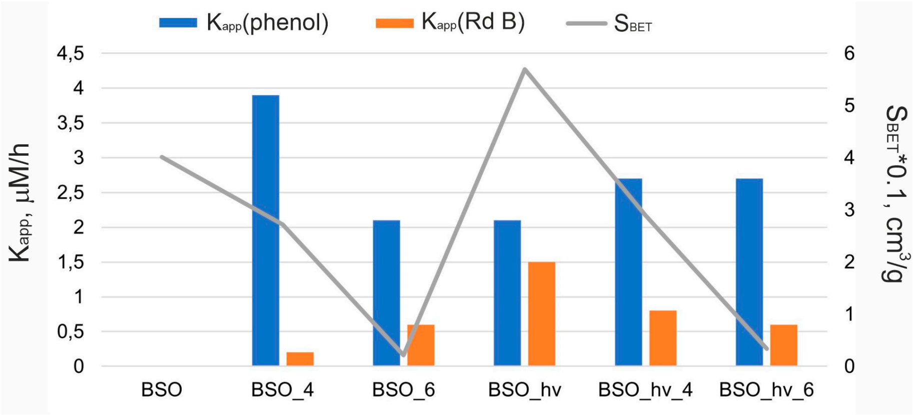

Figure S8. Photodecomposition rate constants difference in comparison with S_{BET} difference for some samples from the BSO and BSO_hv groups.
